# Financing COVID-19 vaccination in sub-Saharan Africa: lessons from a nation-wide willingness to pay (WTP) survey in Ghana

**DOI:** 10.1186/s12889-022-13602-1

**Published:** 2022-06-30

**Authors:** Robert Kaba Alhassan, Edward Nketiah-Amponsah, Mustapha Immurana, Aaron Asibi Abuosi

**Affiliations:** 1 Institute of Health Research, University of Health and Allied Sciences, P.O Box PMB 31, Ho, Ghana; 2 Centre for Health Policy and Implementation Research, Institute of Health Research, University of Health and Allied Sciences, PMB 31, Volta Region Ho, Ghana; 3 Department of Economics, University of Ghana, Legon, P.O Box LG 43, Accra, Ghana; 4 Department of Public Administration and Health Services Management, University of Ghana, Legon, P.O Box LG 78, Accra, Ghana

**Keywords:** Coronavirus disease 2019 (COVID-19), Ghana, Sub-Saharan Africa, Vaccine, Willingness to pay

## Abstract

**Background:**

Over 13 million doses of the corona virus disease, 2019 (COVID-19) vaccines have been administered in Ghana as at March, 2022; 28.5% of the population have received one dose while 16.3% have been fully vaccinated. Cost associated with COVID-19 vaccinations in low- and middle-income countries (LMICs) requires rethinking on sustainable funding arrangements to consolidate gains made towards containing the COVID-19 pandemic.

**Objective:**

Ascertain the determinants of willingness to pay (WTP) for COVID-19 vaccination among adult eligible population in Ghana, and prefer evidence-based policy recommendations on sustainable financing regime for COVID-19 vaccination in the global south.

**Methods:**

*Setting/design:* A cross-sectional web-based survey was conducted among adult population aged 18 years and above across the sixteen (16) administrative regions of Ghana.

*Participants:* A sub-sample of 697 participants willing to receive the COVID-19 vaccine was used as the unit of analysis.

*Outcome measures:* main outcome measures of interests were willingness to pay for COVID-19 vaccination and the specific amount respondents were willing to pay. The odds of WTP and specific amount were predicted using the step-wise backward logistic regression and backward step-wise OLS, respectively.

**Results:**

A total of 2,107 adult respondents aged 18 years and above were reached out to answer the questionnaire; 1,556 successfully completed the questionnaire, representing 74% response rate. Out of the 1,556 valid responses, 697 said they will receive the COVID-19 vaccine. Out of the 697 sub-sample willing to accept the vaccine, 386 (55%) were willing to pay an average of US$6.00 for the vaccine. Positive predictors of WTP were: being an educated male (OR = 0.55, 95% [CI = 0.366, 0.826], *p* = 0.004), married and educated (OR = 2.19, 95% [CI = 1.077, 4.445], *p* = 0.030), being a married health worker (OR = 0.43, 95% [CI = 0.217, 0.845], *p* = 0.015), and having positive perception of the vaccine (OR = 2.40, 95% [CI = 1.144, 5.054], *p* = 0.021). High WTP amounts correlated positively with adherence to COVID-19 prevention protocols (Coef. = 10.30, 95% [CI = 0.463, 20.137], *p* = 0.040) and being a health worker with tertiary education (Coef. = 56.339, 95% [CI = 8.524, 104.154], *p* = 0.021). Christians who are also health workers by occupation were less likely to pay higher amounts for the vaccine (Coef. = -71.431, 95% [CI = 118.821, -24.040], *p* = 0.003).

**Conclusions:**

WTP for COVID-19 vaccination in Ghana is low relative to comparative studies in the sub-region. There is the need for accelerated, advocacy and public education on the benefits of vaccination. Likewise, there should be broader stakeholder engagement and national dialogue on sustainable financing options for COVID-19 vaccination as donor support continues to dwindle for LIMCs like Ghana.

**Supplementary Information:**

The online version contains supplementary material available at 10.1186/s12889-022-13602-1.

## Background

Health care financing remains a critical component of the World Health Organization (WHO) health system building blocks [1]. Unfortunately, lower-middle-income countries (LMICs) with limited resources continue to struggle to finance health care like other sectors of their economies [2].

According to the 2012 National Research Council (NRC), as at 2009, low-income countries (LICs) around the world spent an average of 6.1% of their gross domestic products (GDPs) on health while LMICs, like Ghana, spent 6.2%, and upper-middle-income countries (UMICs) spent 7.0% [3]. Within the sub-Saharan Africa region, an estimated 6.1% of GDP was spent on health in 2009 and later dropped to 5.2% in 2018 [2], less than the 9.5% average for OECD countries [2]. Since 2012, the narrative has remained unchanged in many countries in the global south, including Ghana, which spent 3.5% of its GDP on health in 2018 [2].

In terms of *per capita* expenditure on health, low-income countries spent $25 per person on health in 2009 compared to over $4,600 *per capita* in high-income countries [3]. In the WHO African region, *per capita* health spending was estimated to be $83, less than 2% of the average spending in high-income countries [3].

In Ghana, percentage of budget allocation to the health sector is still below the Abuja Declaration target of 15% [4]. Likewise, health insurance cover for households is less than 40%, resulting in perpetual impoverishment of many citizens due to catastrophic out-of-pocket (OOP) expenditure on health [5, 6]. The incessant rhetoric on attainment of universal health coverage (UHC) will remain illusionary in the foreseeable future if health financing mechanisms for health systems are not re-engineered as a matter of urgency. This call is particularly germane as the world continues to encounter pandemics like the novel coronavirus decease 2019 (COVID-19) with dire consequences for already fragile economies and health systems.

COVID-19 pandemic has worsened the plight of health systems, especially ailing ones in resource-limited settings in Africa. The economic impact of the pandemic has further limited health funding capacities of countries. Moreover, health insurance schemes have suffered from the COVID-19 pandemic because of reduction in donor inflows, reduced employer-based insurance due to high unemployment rates induced by the draconian response strategies against the COVID-19 pandemic by governments [7].

Additionally, the impact of the pandemic on African economies and health financing regimes is evident in the literature [8]. Even though the initial response strategy against the pandemic was centered on non-pharmaceutical interventions (NPIs) such as regular handwashing, social distancing, wearing of face masks and sanitizing, these NPIs remained interim measures until various vaccines were discovered and rolled out. Discovery of vaccines for COVID-19 is therefore widely believed to be a more sustainable long-term approach towards achieving herd immunity and control of the virus [9, 10].

Vaccination has become an effective response strategy against the COVID-19 pandemic with vaccinated persons proven to be less likely to develop serious complications when infected with the virus [11–13]. In light of this, the WHO actively advocates for greater commitment to the vaccine rollout towards attaining herd immunity among populations [14–16].

Globally, as at 5^th^ April 2022, a total of 11,183,087,530 vaccine doses have been administered and approximately 64% of the world’s population had received at least one dose of a COVID-19 vaccine [17, 18]. Unfortunately, a significant proportion of this vaccination coverage is in the western world. COVID-19 vaccination coverage in LMICs is estimated to be around 10.6%. For instance, in Ghana, as at 30^th^ March 2022, a total of 13,163,059 vaccine doses were administered; barely 16% of the eligible population have been fully vaccinated while nearly 29% have received at least one dose of the vaccine [19].

Even though COVID-19 vaccination is currently free with all related cost fully absorbed by states, or through donations, the sustainability of this financing approach is being questioned against the backdrop that the pandemic may linger for a while [20–22]. Many countries in Africa continue to seek donor support from the global north to acquire vaccines for their citizens [23–25]. Coupled with the limited capacity of LMICs to locally produce COVID-19 vaccines, the costs associated with the purchase, transport and last mile distribution of COVID-19 vaccines further compound predicaments of already fragile health systems [25].

Additionally, the increasing acceptance rate of the vaccine, albeit snail-paced, is pointing to a potential funding gap for COVID-19 vaccines [26]. In light of this, many Asian [23, 27] and western countries [28, 29] have initiated the discourse on sustainable funding arrangements for COVID-19 vaccination. Unfortunately, within the context of LMICs, the debate on sustainable financing solutions for COVID-19 vaccination remains muted evidenced by the paucity of empirical research on willingness to pay (WTP) for COVID-19 vaccination in these resource constrained settings.

The need for rethinking on sustainable vaccine financing models in the fight against the COVID-19 is no more optional. This is because of the associated burdensome cost of procuring and administering COVID-19 vaccines, coupled with the fact that GAVI Alliance (formerly the Global Alliance for Vaccines and Immunisation) and other donor partners are gradually weaning off financial and logistical support for many LMICs including Ghana. Even though some authorities argue that the debate on WTP for COVID-19 vaccines might not be ripe [30–32], others hold the strong opinion that it is appropriate to explore and understand WTP for COVID-19 vaccines. Early scientific investigations on the subject matter will inform policy decisions on sustainable financing mechanisms for vaccination campaigns, particularly in resource-limited countries [33–35].

Per the existing financing strategy for vaccines in resource limited settings like Ghana, procurement and supply of vaccines under the Expanded Programme on Immunization (EPI) are funded by donor partners such as the GAVI Alliance, World Health Organization (WHO) and United States Agency for International Development (USAID) to promote Universal Health Coverage (UHC) for vaccine-preventable diseases.

In Ghana, vaccinations of all kinds are not covered by the National Health Insurance Scheme (NHIS) as a primary service, even though the scheme through its UHC agenda contributes a percentage of its revenue towards the procurement, shipment and distribution of vaccines in-country. Indeed, the NHIS Medicines List [36] categorically states that “Medicines used for healthcare programmes are considered as exemptions and are thus excluded from the List [NHIS Medicines List]. These include childhood immunizations, tuberculosis and mental health care” (NHIS Medicines List [36], p 4). This current financing gap presents an opportunity to initiate national policy dialogue, backed by empirical evidence, on the prospects of reviewing the NHIS inclusion list to cover vaccines.

Although some publications exist on this important topic in the developed world, there is paucity of empirical evidence in Africa and Ghana, in particular. This nation-wide web-based survey was conducted from 18^th^ September to 23^rd^ October, 2020. Data was collected few months after China and Russia put out their vaccines in June 2020 and August 2020 respectively. Subsequently, emergency use authorization (EUA) was granted to the first western vaccines in December 2020. Main objective of this study is to ascertain WTP for COVID-19 vaccination among adult eligible populations in all 16 administrative regions of Ghana.

### Theoretical framework

The health belief model (HBM) was adapted as the theoretical basis for conceptualizing WTP for COVID-19 vaccines in the study. According to pioneering works of Rosenstock [37] and Hochbaum [38], HBM explains health seeking behaviour of persons based on socio-economic, demographic, and psychological dynamics. These determinants invariably inform the perceived susceptibility and severity of a health condition on the one hand and the perceived benefits and barriers of a health intervention.

**Fig. 1:**
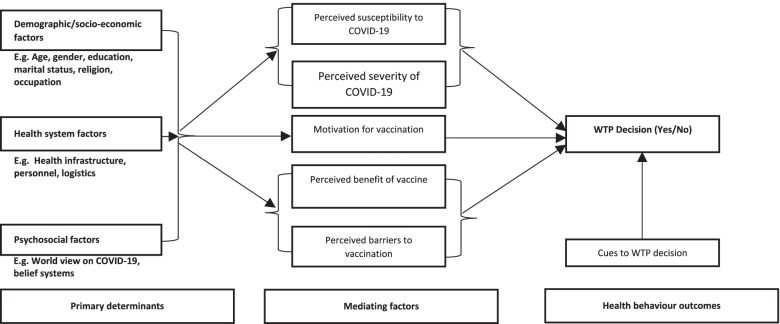
Health belief model adapted for the WTP concept. Source: Conceptualized by authors based on an adapted health belief model by Rosenstock (1974) and Hochbaum (1958)

The HBM concepts were adapted to inform the development of research questions and data collection tools for this study. In the context of this study, the demographic factors are gender and age. Socio-economic factors are occupation, marital status, religion and level of education. Mediating factors conceived under the HBM are perceived susceptibility to contracting COVID-19 and suffering severity, intrinsic motivation for vaccination, perceived benefits and barriers to taking COVID-19 vaccination. The expected behaviour outcome is conceptualized to be a decision to pay or not pay for the COVID-19 vaccine. The HBM was further adapted to include some health system level factors such as availability of health infrastructure, personnel and logistics (see Fig. 1).

It must be acknowledged that due to the focus of this paper, the health system level factors were not all explored in detail as determinants of WTP behaviour. Additionally, WTP decisions are quite complex and possibly transcend the conceptualized primary determinants and mediating factors outlined in the adapted HBM. This gap therefore constitutes a potential limitation of the model in the context of this paper and it is duly acknowledged.

## Methods

### Study design

 A cross-sectional web-based survey was conducted across the sixteen (16) administrative regions of Ghana prior to deployment of the first batch of COVID-19 vaccines in the country.

### Study setting/population

The study was conducted among adult populations aged eighteen (18) years and above on their willingness to pay for COVID-19 vaccination. The researchers used the Population and Housing Census (PHC) estimate of 30 million Ghanaian population as the proxy target population and derived the representative sample size using Krejcie and Morgan’s [39] formula for calculating sample size based on known population. Based on this formula, a sample size of 1500 was deemed adequate at 95% confidence level. This sample size determination was used in earlier publications by the authors [40, 41].

### Sampling procedure

Non-probability sampling technique was used where the questionnaires were conveniently administered to eligible respondents who were willing to participate in the study. Even though this sampling procedure has limitation of potential self-selection bias, the large representative sample size compensated for this potential limitation.

### Instruments of data collection

The questionnaire for data collection was hosted on the REDCap platform (developed by Vanderbilt University in Nashville, Tennessee USA). As a result of restrictions on human movement and contact due to the COVID-19 pandemic, the questionnaires were predominantly distributed via social media platforms and other electronic networks. Trained research assistants were also used to promote and follow up on respondents by way of polite reminders. All respondents granted voluntary consent to participate before they accessed the questionnaire content.

Instances where target participants could not access the online tool due to illiteracy or non-ownership of smartphones, they were assisted to answer the questions in-person by trained research assistants under strict COVID-19 preventive measures.

Research assistants who were already resident in the respective 16 administrative regions were recruited and given online training for the complimentary in-person data collection. The tool was read and translated into the local language during the in-person interviews. It must however be conceded that due to the COVID-19 restrictions, the in-person complimentary data collection was practicable on some occasions and did not represent the generality of the method used for the data collection.

The data collection instrument comprised of open and close ended questions categorized into sections of socio-demographic characteristics; views on COVID-19 and the vaccine including willingness to pay for it. Cronbach’s *alpha* test for internal reliability found the average scale reliability coefficient to be above the 80% rule of thumb (see Supplementary File 1).

### Ethical considerations

Ethical clearance was sought from the Research Ethics Committee (REC) of the University of Health and Allied Sciences, Ghana (clearance number: UHAS-REC A.1[6] 20–21). Only respondents who provided voluntary informed consent were able to access the online questionnaire and later submit data as part of the study. Moreover, responses were all coded for anonymity.

### Patient and public involvement

No patient was involved in this study. However, the research questions and outcome measures were informed by respondents’ priorities, experiences, and preferences. Moreover, through previous engagements with relevant stakeholders, respondents’ responses were fed into the design of this study. Also, all respondents who volunteered to participate in the study were actively engaged throughout the study through pre-tests and follow-ups. Finally, community durbars and advocacy meetings are part of the dissemination plans with the relevant stakeholders to inform policy.

### Data analysis

Data was analysed using STATA statistical analysis software (StataCorp. 2011, Stata Statistical Software: Release 12. College Station, TX: StataCorp LP) after cleaning and coding. Guided by the HBM constructs, the main dependent variables of interest were: whether or not respondents will pay for COVID-19 vaccination (yes = 1; no = 0), and how much in Ghana Cedis (GHC) they are willing to pay (continuous). Independent variables of interest were: the socio-demographic factors such as sex (male = 0; female = 1), age (≥ 48 years = 1; < 48 years = 0), marital status (not-married = 0; married = 1), occupation (health worker = 1, other = 0), region (Greater Accra = 1; other = 0), education (No formal education = 1; formal education = 0); and religion (Christian = 1; other = 0). These independent variables were further interacted among themselves to ascertain their mutual effect on the main outcome variables of interest.

Additionally, five (5) indexed explanatory variables of interest were created as proxies for overall perception of COVID-19, adherence to COVID-19 protocols, perceived impact of COVID-19 on livelihood, satisfaction with government response strategies, and perception of the COVID-19 vaccine.

Chi-square and Fisher’s Exact tests were used to test for differences in categorical variables between two sub-groups at 95% confidence level. The backward step-wise logistic (BSL) regression analysis was used to ascertain the odds of WTP for COVID-19 vaccination. The BSL regression involved starting with a full model (model 1) with 19 independent variables and iterated down to twelve (12) variables after omitting collinear explanatory variables. Likewise, an OLS regression analysis was conducted (model 2). This OLS model was fitted with 19 variables and iterated backwards. Six (6) non-collinear predictor variables were retained to ascertain determinants of the exact amount respondents were willing to pay for COVID-19 vaccination.

## Findings

### Socio-demographics

A total of 2,107 adult respondents aged 18 years and above were reached out to answer the questionnaires; 1,556 successfully completed the questionnaires, representing 74% response rate. Out of the 1,556 valid responses, 697 said they will receive the COVID-19 vaccine; 55% of the 697 respondents expressed willingness to pay for the vaccine. Since this paper is focused on WTP for the COVID-19 vaccination, the sub-sample of 697 complete records was used as the unit of analysis.

The results further show that 54% of the respondents were males, the age group was largely youthful with a mean age of 33; 88% of them were within 18–41 years; close to 68% of the respondents had at least tertiary education; approximately 45% of the respondents were employed in the formal sector while most of them were resident in Greater Accra (25%) and Volta (23%) regions; nearly 45% of respondents were married while 90% were Christians.

Out of the total number of 697 respondents willing to accept vaccination, 386 (55%) of them were willing to pay an average amount of Ghana Cedis (GHC) 38.00 (approximately US$ 6.00^1^) (see Table 1).

**Table 1:** Background characteristics of respondents

Socio-demographic characteristics	Statistics	
**Sex**	**Freq. (f)**	**Percent (%)**
Male	375	54.35
Female	315	45.65
**Total**	**690**	**100.00**
**Age range**		
18–23	99	15.28
24–29	160	24.69
30–35	208	32.10
36–41	100	15.43
42–47	37	5.71
48 and above	44	6.79
**Total**	**648**	**100.00**
**Average age** (years) (Obs. = 628; mean, Std. Dev.)	32.70	10.091
**Education**		
No formal education	105	15.33
Primary	22	3.21
Middle/JSS/JHS	21	3.07
Secondary (SSS/SHS)	68	9.93
Tertiary	469	68.47
**Total**	**685**	**100.00**
**Occupation**		
Artisan	29	4.30
Farmer	50	7.42
Teacher	135	20.03
Health worker	172	25.52
Trader	109	16.17
Other (please specify)	179	26.56
**Total**	**674**	**100.00**
**Region**		
Ashanti Region	36	5.32
Ahafo Region	5	0.74
Brong-Ahafo Region	11	1.62
Bono-East Region	17	2.51
Central Region	75	11.08
Eastern Region	74	10.93
Greater Accra Region	172	25.41
Northern Region	26	3.84
Oti Region	2	0.30
Upper East Region	12	1.77
Upper West Region	21	3.10
Volta Region	156	23.04
Western Region	58	8.57
Western-North Region	12	1.77
**Total**	**677**	**100.00**
**Marital status**		
Divorced	16	2.43
Living together	29	4.41
Married	293	44.53
Never married	301	45.74
Separated	13	1.98
Widowed	6	0.91
**Total**	**658**	**100.00**
**Religious affiliation**		
Christian	621	90.39
Moslem	53	7.71
Traditionalist	9	1.31
Other (specify)	4	0.58
**Total**	**687**	**100.00**
**Will pay for COVID-19 vaccine**		
No	311	44.88
Yes	382	55.12
**Total**	**693**	**100.00**
**Average amount (GHC) willing to pay** (Obs. = 319, Mean, Std. Dev.)	37.80	65.50

Source: Field Survey, 2020

**Fig. 2:**
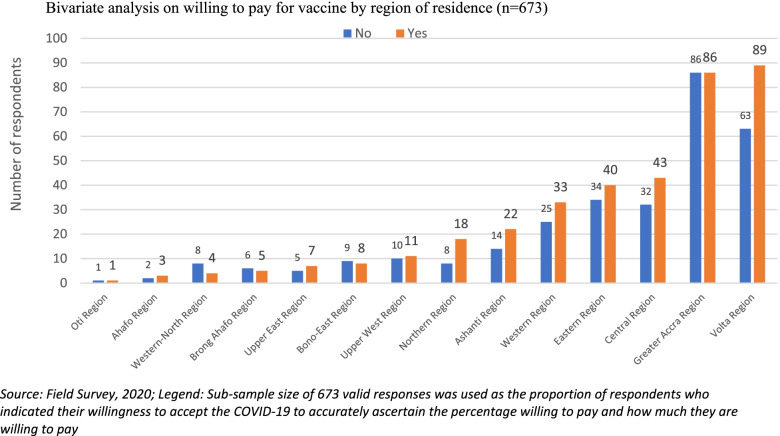
Bivariate analysis on willing to pay for vaccine by region of residence (*n* = 673). Source: Field Survey, 2020; Legend: Sub-sample size of 673 valid responses was used as the proportion of respondents who indicated their willingness to accept the COVID-19 to accurately ascertain the percentage willing to pay and how much they are willing to pay

**Fig. 3:**
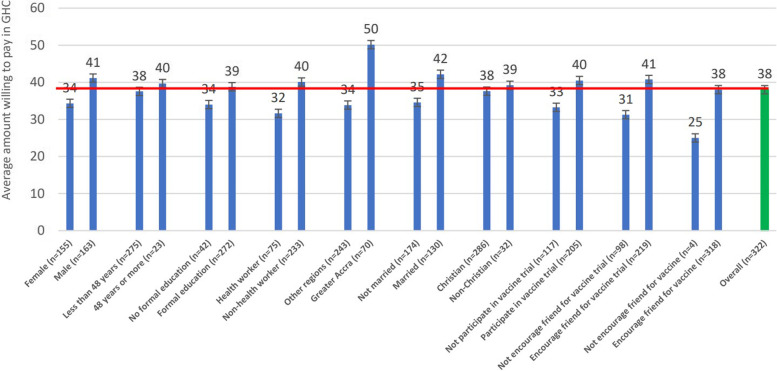
Bivariate analysis on average amount (GHC) willing to pay for vaccine and associated factors. Source: Field Survey, 2020

**Fig. 4:**
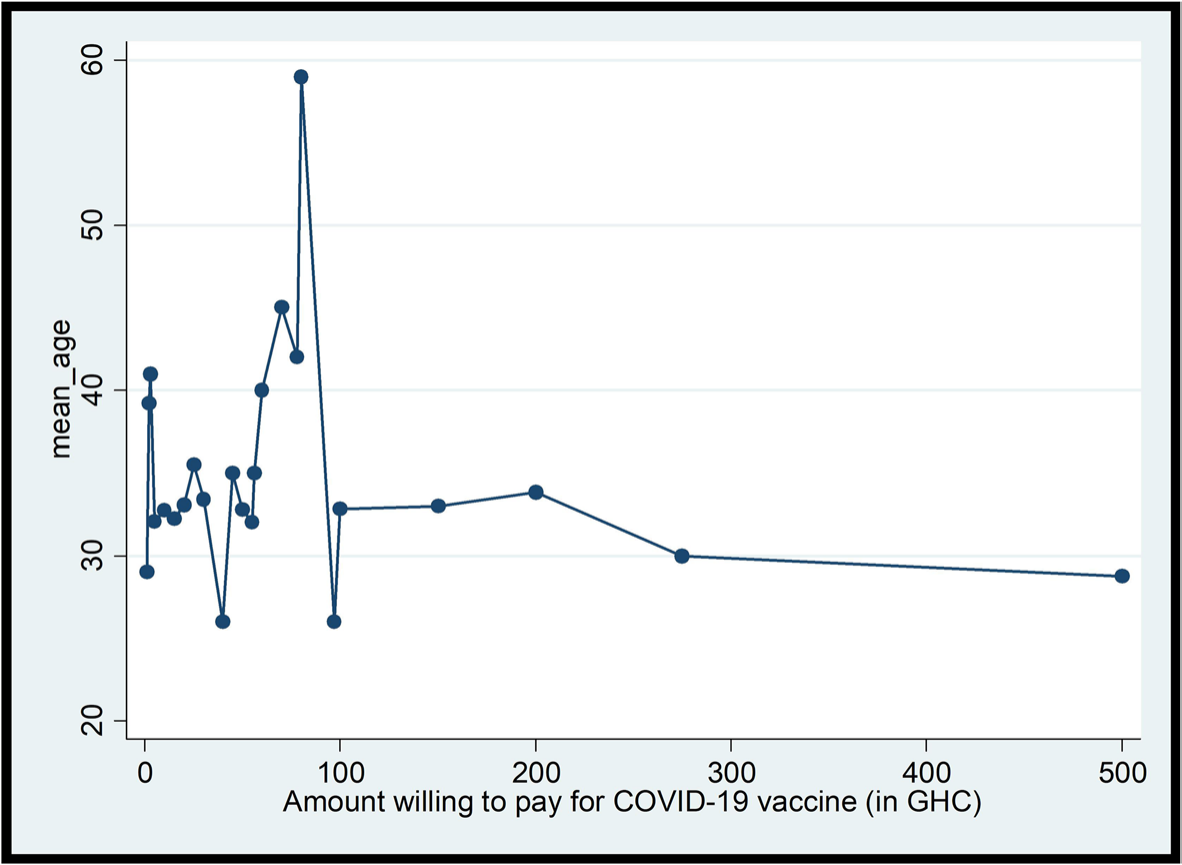
Correlation between mean age and amount willing to pay for COVID-19 vaccine. Source: Field Survey, 2020

**Fig. 5:**
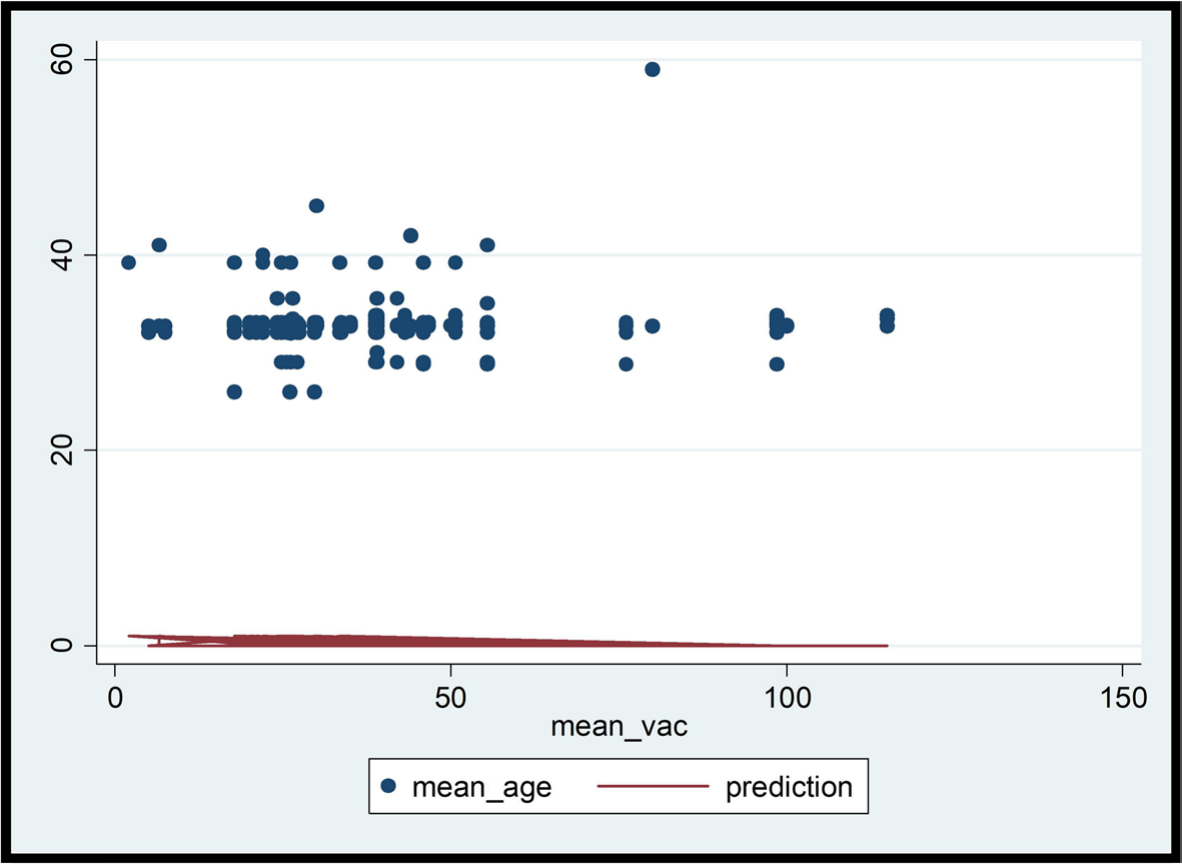
Prediction of amount willing to pay for COVID-19 vaccine based on mean age. Source: Field Survey, 2020

Bivariate Chi-square and Fisher’s Exact analyses were conducted on the factors associated with willingness to pay for the COVID-19 vaccination. As shown in Table 2, more males and persons who have at least tertiary education indicated their willingness to pay for COVID-19 vaccination, albeit statistically insignificant. Similarly, persons who said they will participate in a COVID-19 vaccine trial or accept the vaccine and recommend it to others were more willing to pay for the vaccination (see Table 2). Respondents who were resident in Greater Accra and Volta regions expressed willingness to pay for vaccination than their counterparts in other regions (see Fig. 2). Residents in Greater Accra region were willing to pay more for the vaccination, relative to residents in other regions (see Fig. 3). However, there was an inverse relationship between age and WTP amounts; thus, WTP amount reduces as age of respondent increases (see Figs. 4 and Fig. 5).

**Table 2:** Bivariate analysis on socio-demographics and willingness to pay for COVID-19 vaccine

Characteristics	Statistics			*p*-value
	**WTP Decision**			
	**No**	**Yes**	**Total**	
**Sex**	**Freq. (%)**	**Freq. (%)**	**Freq. (%)**	
Male	183 (26.26)	194 (27.83)	377 (54.09)	0.055**
Female	135 (19.37)	185 (26.54)	320 (45.91)	
**Total**	**318 (46.62)**	**379 (54.38)**	**697 (100.00)**	
**Age range**				
18–23	46 (7.03)	56 (8.56)	102 (15.60)	0.933*
24–29	68 (10.40)	93 (14.22)	161 (24.62)	
30–35	99 (15.14)	112 (17.13)	211 (32.26)	
36–41	47 (7.19)	53 (8.10)	100 (15.29)	
42–47	18 (2.75)	19 (2.91)	37 (5.66)	
48 and above	18 (2.75)	25 (3.82)	43 (6.57)	
**Total**	**296 (45.26)**	**358 (54.74)**	**654 (100.00)**	
**Education**				
No formal education	52 (7.51)	53 (7.66)	105 (15.17)	0.504*
Primary	13 (1.88)	9 (1.30)	22 (3.18)	
Middle/JSS/JHS	7 (1.01)	13 (1.88)	20 (2.89)	
Secondary (SSS/SHS)	31 (4.48)	39 (5.64)	70 (10.12)	
Tertiary	212 (30.64)	263 (38.01)	475 (68.64)	
**Total**	**315 (45.52)**	**377 (54.48)**	**692 (100.00)**	
**Occupation**				
Artisan	16 (2.35)	14 (2.06)	30 (4.41)	0.398*
Farmer	27 (3.96)	23 (3.38)	50 (7.34)	
Teacher	56 (8.22)	82 (12.04)	138 (20.26)	
Health worker	85 (12.48)	87 (12.78)	172 (25.26)	
Trader	50 (7.34)	61 (8.96)	111 (16.30)	
Other (please specify)	77 (11.31)	103 (15.12)	180 (26.30)	
**Total**	**311 (45.67)**	**370 (54.33)**	**681 (100.00)**	
**Marital status**				
Divorced	5 (0.75)	11 (1.65)	16 (2.41)	0.490*
Living together	14 (2.11)	15 (2.26)	29 (4.36)	
Married	146 (21.95)	150 (22.56)	296 (44.51)	
Never married	134 (20.15)	172 (25.86)	306 (46.02)	
Separated	4 (0.60)	8 (1.20)	12 (1.80)	
Widowed	2 (0.30)	4 (0.60)	6 (0.90)	
**Total**	**305 (45.86)**	**360 (54.14)**	**665 (100.00)**	
**Religion**				
Christian	291 (41.81)	338 (48.56)	629 (90.37)	0.228*
Moslem	18 (2.59)	36 (5.17)	54 (7.76)	
Traditionalist	5 (0.72)	4 (0.57)	9 (1.29)	
Other (specify)	1 (0.14)	3 (0.43)	4 (0.57)	
**Total**	**315 (45.26)**	**381 (54.74)**	**696 (100.00)**	

Source: Field Survey, 2020

^*^Fisher’s Exact test at 95% significance level

^**^1-sided Fisher’s Exact test 95% significance level

### Correlates of WTP for COVID-19 vaccine

Results from the backwards step-wise regression analysis showed that the odds of paying for COVID-19 vaccination increases with being an educated male (OR = 0.55, 95% [CI = 0.366, 0.826], *p* = 0.004), having positive mindset/perception of the vaccine (OR = 2.40, 95% [CI = 1.144, 5.054], *p* = 0.021), being married and educated (OR = 2.19, 95% [CI = 1.077, 4.445], *p* = 0.030) and being a married health worker (OR = 0.43, 95% [CI = 0.217, 0.845], *p* = 0.015), after controlling for co-variates (see Table 3).

**Table 3:** Backwards stepwise regression on predictors of willingness to pay for COVID-19 vaccine (*n* = 559)

Willingness to pay	OR	Std.Err	*P* > z	[95%Conf	Interval]
Elderly male	0.199	0.225	0.153	0.022	1.820
Educated male	0.550	0.114	0.004	0.366	0.826
Adherent to COVID-19 protocols^a^	0.887	0.107	0.320	0.700	1.123
Married Christian	0.549	0.173	0.057	0.296	1.018
Perception of vaccine^b^	2.404	0.911	0.021	1.144	5.054
Educated elderly	3.178	3.738	0.326	0.317	31.871
Elderly married	5.205	4.892	0.079	0.825	32.844
Elderly Christian	0.373	0.290	0.204	0.082	1.709
Response to COVID-19^c^	1.171	0.123	0.134	0.952	1.439
Educated married	2.188	0.791	0.030	1.077	4.445
Elderly Christian	1.422	0.325	0.124	0.908	2.225
Married health worker	0.428	0.149	0.015	0.217	0.845
_cons	0.573	0.298	0.285	0.207	1.589

*Legend*: ^a^indexed score of overall adherence level to COVID-19 protocols on a five-point Likert scale *where* higher values depict better adherence and vice-versa; ^b^indexed score of perception of COVID-19 where higher values depict positive perception and vice-versa; ^c^indexed score on perceived government response strategy against COVID-19 on a five-point Likert scale where higher values depict better perceived response and vice-versa

*Note*: Sample size (*n* = 559) in the regression model is the valid responses of persons who will accept to take the COVID-19 vaccine and those who did not respond in the affirmative were dropped from the regression model

**Step-wise backwards regression beginning with full model;**

*p* = 0.9929 >  = 0.3300 removing Male Christian

*p* = 0.9461 >  = 0.3300 removing Educated health worker

*p* = 0.9266 >  = 0.3300 removing Male health worker

*p* = 0.8447 >  = 0.3300 removing Perceived impact of COVID-19

*p* = 0.6085 >  = 0.3300 removing Perception of COVID-19

*p* = 0.5769 >  = 0.3300 removing Christian health worker

*p* = 0.4735 >  = 0.3300 removing Married male

**Logistic regression (model specification)**

Number of obs. = 559

LR chi2(12)= 31.99

Prob > chi2 = 0.0014

Log likelihood = -369.81995

Pseudo R2 = 0.0415

In respect of determinants of the exact amount an adult respondent is willing to pay, the backwards step-wise regression analysis output revealed that willingness to pay for higher amounts for the vaccination corresponded positively with persons who adhered to COVID-19 prevention protocols (Coef. = 10.30, 95% [CI = 0.463, 20.137], *p* = 0.040) or health workers who had at least tertiary educational qualification (Coef. = 56.339, 95% [CI = 8.524, 104.154], *p* = 0.021). However, health workers who were Christians demonstrated a lesser likelihood of paying higher amounts for the vaccine (Coef. = -71.431, 95% [CI = 118.821, -24.040], *p* = 0.003), relative to their comparators, after controlling for the effect of co-variates (see Table 4).

**Table 4:** Backwards stepwise regression on predictors of amount willing to pay for COVID-19 vaccine (*n* = 255)

Amount willing to pay (GHC)	Coef	Std.Err	P > t	[95%Conf	Interval]
Government response to COVID-19^a^	-5.233	4.729	0.270	-14.548	4.082
Elderly married	18.913	18.563	0.309	-17.648	55.473
Adherent to COVID-19 protocol^b^	10.300	4.995	0.040	0.463	20.137
Educated health workers	56.339	24.277	0.021	8.524	104.154
Christian health workers	-71.431	24.061	0.003	-118.821	-24.040
Impact of COVID-19^c^	5.541	4.675	0.237	-3.666	14.748
_cons	2.612	19.077	0.891	-34.962	40.186

*Legend*: ^a^indexed score on perceived government response strategy against COVID-19 on a five-point Likert scale where higher values depict better perceived response and vice-versa; ^b^indexed score of overall adherence level to COVID-19 protocols on a five-point Likert scale where higher values depict better adherence and vice-versa; ^c^indexed score on perceived impact of COVID-19 livelihood on a five-point Likert scale where higher values depict perceived less impact and vice-versa

*Note*: Sample size (*n* = 255) in the regression model is the valid responses of persons who will accept to pay for the COVID-19 vaccine and those who did not respond in the affirmative were dropped from the regression model

**Step-wise backwards regression beginning with full model**

*P *= 0.9986 >= 0.3300 removing Married male

*P* = 0.9866 > = 0.3300 removing Perception of COVID-19

*p* = 0.9673 > = 0.3300 removing Elderly educated

*p* = 0.9315 > = 0.3300 removing Married health worker

*p* = 0.9022 > = 0.3300 removing Married Christian

*p* = 0.7673 > = 0.3300 removing Elderly male

*p* = 0.7735 > = 0.3300 removing Elderly Christian

*p* = 0.5059 > = 0.3300 removing Male health worker

*p* = 0.4332 > = 0.3300 removing Educated married

*p* = 0.5386 > = 0.3300 removing Educated Christian

*p* = 0.3999 > = 0.3300 removing Perception of vaccine

*p* = 0.4132 > = 0.3300 removing Male Christian

*p* = 0.5735> = 0.3300 removing Educated male

**Ordinary Least Squares Regression (Model specification)**

Source SS df MS Number of obs. = 255

Model 73,041.375 6 12173.563 Prob > F = 0.005

Residual 9.62e + 05 248 3879.462 R-squared = 0.071

Total 1.04e + 06 254 4075.386 Root MSE = 62.285

## Discussion

Findings from the study indicate that generally, 55% of persons who will accept vaccination against COVID-19 were also willing to pay for the vaccine. This percentage is lower than the 78% acceptance rate in a similar study in Indonesia [31] but a little higher than the acceptance rate of 51% recorded among persons aged 15 years and above in an earlier study conducted on Ghana [42]. Even though the findings in Acheampong et al. [42] are consistent with the current study in terms of vaccine acceptance, the study did not highlight WTP for the COVID-19 vaccine. In similar studies outside Ghana, Sarasty et al. [43] found acceptance rate of the vaccine to be approximately 97% and WTP rate to be 85% in Ecuador, which is higher than the 48% acceptance rate recorded in Malaysia [44]. In Africa, Ilesanmi et al. [33] observed that WTP for COVID-19 vaccine in Nigeria was 62%, higher the 55% acceptance rate recorded in this study. Perhaps timing of the two studies and the country-specific dynamics could account for these differences.

This current study found that the average amount a respondent is willing to pay for the vaccine was approximately US$ 6.00 compared to a similar study in Ecuador where conservative estimates of the average WTP values ranged from US$ 147.61 to 196.65 [43] relative to US$184 in Chile [45], between US$23 and US$11.5 in Malaysia [44], US$14–72 in China [46] and US$13.16 in Nigeria [33]. The differences in the WTP average values might be attributed to the economic status of the countries and average *per capita* expenditure on health by these countries. With the exception of Nigeria, the *per capita* expenditure on health in Ghana is relatively lower than the other countries and could explain these WTP dynamics.

Nguyen et al. [47] found in their study that 82.6% of the 651 pregnant women surveyed expressed an average WTP amount of US$ 15.2 ± 27.4. Nonetheless, Nguyen et al. [47] mainly concentrated on pregnant women making their findings not suitable for comparison with this current study which focused on the general adult population aged 18 years and above. Future studies on targeted vulnerable populations like pregnant women in Ghana could help understand the WTP decisions among these populations with unique health needs even though there is currently a free maternal health care policy under the NHIS.

In terms of the determinants of specific WTP amount, Sarasty et al. [43] observed that duration of protection of the vaccine significantly influenced WTP decisions by citizens. Conversely, Nguyen et al. [47] found that higher income, having children, self-perceived risk of COVID-19 infection, and perceived risk to friends were significantly associated with a higher likelihood of accepting and paying for the COVID-19 vaccine.

These findings affirm the constructs in earlier studies [37, 38] on the HBM which argued that perceived susceptibility and severity of a disease; perceived benefits and barriers to accessing a health intervention significantly influence health seeking behaviours including WTP for the COVID-19 vaccine. In light this, interventions aimed at promoting WTP among populations should prioritise these important determinants. These determining factors then ought to be addressed through effective stakeholder consultations and engagement.

Moreover, this study found a positive association between WTP and higher educational qualification, adherence to COVID-19 preventive measures and being a health worker. These observations are similar to findings on Malaysia [41], Chile [44], Nigeria [33] and some Asian countries [43, 44, 46]. The findings also corroborate HBM constructs on the primary determinants of health seeking behaviour such as socio-economic and demographic factors [37, 38]. On the contrary, Ilesanmi et al. [33] observed that the significant predictor of WTP among 440 community members in Ibadan Nigeria was the need to stay healthy while unwillingness to pay for the vaccine was attributed to households’ inability to afford the cost [33].

In view of these revelations, persons within the lower socio-economic bracket should be targeted in future fee exemption policies for vaccinations including the COVID-19 vaccine. Previous studies have established the association between health behaviour and socio-economic factors within the framework of social determinants of health [48]. Findings in this study therefore corroborate the postulations in Marmot et al. [48].

Dynamics on the determinants of WTP for the COVID-19 vaccine appeared to be similar across the various countries in Africa, except that the average WTP amounts varied according to economic situation in the pertinent countries. For instance, in Kenya, a WTP study found that approximately 80% of 1,050 study participants were willing to pay between US$49.81 and US$68.25 [35]. Significant predictors of WTP in the Kenyan study were: vaccine duration of protection and efficacy, perceived probability of being hospitalized, age, gender, education, location (region of residence), and household income [35]. These observations corroborate the findings in this current study and the HBM constructs on predictors of health seeking behaviour of individuals and households [37, 38].

Some findings in this study are however at variance with similar studies conducted in some African countries. These variances could be attributed to a number of factors. For instance, nearly 68% of the respondents who participated in this study had at least tertiary education and a significant percentage were either health workers (24%) or teachers (20%). The hypothesis is that these categories of respondents are more likely to have better appreciation of the benefits of the vaccine and perhaps more likely to spare higher amounts to pay for the vaccine as explained by Carpio et al. [35], Acheampong et al. [42], Sarasty et al. [43] and the HBM [37, 38].

Additionally, the variable “religion” when fitted independently in the model did not significantly predict WTP decisions but was later interacted with the independent variable “occupation”. Following the variable interactions, it was found that Christians who are also health workers were less likely to spend higher amount for the COVID-19 vaccine. This observation appears to be counter-intuitive because, the combine effect of being a health worker and belonging to a religious faith like Christianity is expected to have favoured the odds of willingness to pay more for the vaccine as argued in other studies [35, 42, 43]. The authors however, concede that the results could be attributed to the interactive effect of the variables: “religion” and “occupation” on WTP since they were merged into a single independent variable in the regression model.

The findings are nonetheless consistent with outcomes of similar studies which demonstrated that religion is a strong predictor of perceptions and uptake of health interventions including the COVID-19 vaccine [9]. On the other hand, the findings from the current study might reflect a reality that persons of higher socio-economic status expect more from state institutions. In effect, citizens' social contract with their governments includes taking care of their health needs, including funding COVID-19 vaccination. Even though there are no comparative studies to prove or contradict these observations, future studies that employ mixed-methods approach would help unearth this subject matter in detail.

Besides religion and occupation, other significant predictors of WTP for COVID-19 vaccine were level of education, age and marital status. It was found that males who have formal education were more likely to pay for the vaccine, similar to the findings in Malaysia [44], Chile [30], Nigeria [33] and some Asian countries [43, 44, 46]. The patriarchal nature of many societies in Africa and the fact that males turn to be more gainfully employed than females might account for these gender dynamics in the WTP decisions.

Furthermore, the results showed that married persons who are also educated or health workers by profession were more likely to express willingness to pay for the COVID-19 vaccine than their comparators. Primary determinants of the HBM [37, 38] also elucidate the importance of socio-economic factors in health seeking behaviours of individuals. Finally, it was observed that respondents who demonstrated positive mindset on the COVID-19 prevention protocols and the vaccine itself expressed a greater likelihood of paying for the vaccine. These mediating factors under the HBM [37, 38] indeed have been confirmed to significantly influence important health seeking behaviours like WTP decisions. Policy decisions towards co-funding of COVID-19 vaccination must therefore take into account these important mediating factors to guarantee acceptance of such interventions.

## Conclusion

Willingness to pay for COVID-19 vaccine in Ghana was found to be approximately 55%. Similar studies show that WTP varies in sub-Saharan Africa (SSA) and other developing countries, ranging from a low of 48% in Malaysia to a high of 85% in Ecuador. Similarly, the average WTP amount varied among SSA and other developing countries, ranging from US$6.00 in Ghana to a range of US$147.61–196.65 in Ecuador.

Significant predictors of WTP and associated amounts were: level of education, occupation, religious affiliation and perceptions on the COVID-19 vaccine and its safety. These dynamics are important considerations in any discourse on sustainable financing regime for COVID-19 vaccination in the global south. This study is the first of its kind in Ghana and has adduced compelling empirical evidence on WTP. Findings are expected to ignite a national dialogue on sustainable funding mechanisms for COVID-19 vaccination in Ghana as donor support dwindles.

## Limitations

This study was conducted in the latter part of 2020 before the deployment of COVID-19 vaccination in Ghana. This data collection window could have influenced the responses of participants because there was greater uncertainty and fear of the COVID-19 pandemic at that time. Prior to the deployment of the COVID-19 vaccine in many countries, including Ghana, the skepticism and uncertainty on the vaccine was higher and this low confidence levels might have impacted the WTP outcomes. Post-vaccine deployment surveys on WTP might reveal varied findings. Perhaps persons who received the vaccine with positive experience could demonstrate a high preponderance of WTP with higher associated amounts.

Moreover, it is important to acknowledge that the study was a web-based survey which might have exposed it to a potential self-selection bias. Thus, respondents who participated in the study might be persons who predominantly owned a smartphone and are internet savvy. Nonetheless, trained research assistants administered the survey questionnaires to illiterates and persons who didn’t own a smartphone as a strategy to address the potential self-selection bias.

## Policy recommendations

While acknowledging the above limitations associated with this study, the authors propose the following recommendations:

1. National dialogue on allocation of a proportion of the COVID-19 levy in Ghana and countries with similar tax for vaccine-specific funding to help avert future financing constraints when weaned off donor support for vaccines procurement and distribution.
2. Increased public education and awareness creation on benefits of the COVID-19 vaccination will enhance citizens’ confidence and promote WTP for the vaccine when the need arises.
3. Active engagement of religious bodies will help promote public confidence in the COVID-19 vaccine to stimulate high WTP levels

## Supplementary Information

**Additional file 1. **Structured questionnaire: community level.

## Data Availability

All data generated or analyzed during this study are included in this published article and its supplementary information files.

## Footnotes

1 Exchange rate based on 1 US$ = GHC 5.8 as 23.^rd^ October, 2020 (Source: https://www.oanda.com/currency-converter/en/?from=USD&to=GHS&amount=1).
