# Supplementary material for: Financing COVID-19 vaccination in sub-Saharan Africa: lessons from a nation-wide willingness to pay (WTP) survey in Ghana
Source: BMC Public Health. 2022 Jun 30;22:1273. doi: 10.1186/s12889-022-13602-1 (PMC9243858; doi:10.1186/s12889-022-13602-1)
Supplement: Supplementary file 1 — Additional file 1. Structured questionnaire: community level. [file 12889_2022_13602_MOESM1_ESM.pdf]

Structured Questionnaire: Community Level

Preamble and informed consent

Dear Respondent,

We are researchers from the Institute of Health Research, University of Health and Allied Sciences, Ghana and we wish to engage you briefly on your views and experiences with the COVID-19 pandemic in Ghana. You would be specifically asked questions on your knowledge of the COVID-19 pandemic, your views on the preventive measures put in place to prevent and control spread of the disease and your level of compliance with these measures. Additionally, we will ask questions on your views on the impact of government control measures such as lockdown, mandatory wearing of face masks, and social distancing. This exercise is mainly for academic purposes and no personal information is requested from you. Moreover, privacy and confidentiality of the process is highly guaranteed. Your responses will not be linked up to any personal information throughout the survey. The questionnaire will take approximately 5-10 minutes of your time.

If you are satisfied with the participant information and voluntarily agree to participate in this survey, please proceed to click "Yes, I voluntarily agree to participate".

Yes, I Agree to participate [ ] ***No, I am not interested [ ]***

Community/household questionnaire

	SECTION A: Background information/socio-demographics	Response
1	Sex<br>• Male_______________1<br>• Female_______________2	[ ]
2	Actual age (Please specify) _______________	
3	Age range<br>• 18-23__________1<br>• 24-29__________2<br>• 30-35__________3<br>• 36-41__________4<br>• 42-47__________5<br>• 48 and above__________6	[ ]
4	Highest educational qualification<br>• No formal education_______________1<br>• Primary _______________ 2<br>• Middle/JSS/JHS_______________3<br>• Secondary (SSS/SHS) _______________4<br>• Tertiary _______________5	[ ]
5	Current main occupation<br>• Artisan_______________1<br>• Farmer_______________2<br>• Health worker_______________3	[ ]

	• Teacher____4<br>• Trader____5<br>• Other (please specify) ___________________________	
6	Current region of residence<br>• Please specify____	
7	Marital status<br>• Divorced____1<br>• Living together____2<br>• Married____3<br>• Never married____4<br>• Separated____5<br>• Widowed ____6	
8	Religious affiliation<br>• Christian____1<br>• Moslem____ 2<br>• Traditionalist____ 3<br>• Other (specify)____	
	**SECTION B: Knowledge and awareness of COVID-19**	**Response**
1	Coronavirus disease also called COVID-19 is caused by a virus<br>• Yes_____________________________________ 1<br>• No______________________________________2	
2	What is the period between getting infected with the COVID-19 and showing signs and symptoms (incubation period)?<br>• 6 days or less_____________________________________ 1<br>• 7-14 days____2<br>• 15-21 days_______________________________________3<br>• Over 21 days______________________________________4	
3	COVID-19 is more severe and deadly for those with weakened immunity, the elderly and very young<br>• Yes_____________________________________ 1<br>• No______________________________________2<br>• Don't know_______________________________3	
4	Transmission of COVID-19 from one person to another happens when someone comes into contact with infected person's secretions/body fluids (e.g. saliva, aerosols etc.)<br>• Yes_____________________________________ 1<br>• No______________________________________2	
5	Which of these can expose you or someone to COVID-19?<br>**(You can choose more than one)**<br>• Coughing____________________________________ 1<br>• Sneezing_____________________________________2<br>• Handshakes with infected persons______________3<br>• Touching infected surfaces___________________4<br>• Contacts with body fluids of infected persons________5	
6	Which of the following may suggest someone has COVID-19?<br>**(You can choose more than one)**<br>• Bodily pains____1<br>• Easy fatigue____2	

	• Fever____________________________________3<br>• Headache__________________________________4<br>• Loss of smell______________________________5<br>• Loss of taste______________________________6<br>• Persistent cough___________________________7<br>• Runny nose________________________________8<br>• Shortness of breath_________________________9<br>• Sneezing _________________________________10<br>• Sore throat_______________________________11	
7	Which of these would you do **first** if you notice you are showing symptoms of COVID-19?<br>**(Choose only one option)**<br>• Go to hospital____________________________1<br>• Go to chemical shop/pharmacy_______________2<br>• Call the police___________________________ 3<br>• Call COVID-19 response team number__________4<br>• Not disclose______________________________ 5	☐
8	Which of these would you do **first** if you notice a relative/friend/co-worker shows symptoms of COVID-19?<br>**(Choose only one option)**<br>• Take to hospital___________________________1<br>• Take to chemical shop/pharmacy______________2<br>• Call the police___________________________ 3<br>• Call COVID-19 response team number__________4<br>• Not disclose______________________________ 5	☐
9	COVID-19 has a vaccine<br>• Yes_________________________ 1<br>• No__________________________2	☐
10	COVID-19 has a cure<br>• Yes_________________________ 1<br>• No__________________________2	☐
11	How did you **first** hear of COVID-19?<br>**(Choose only one option)**<br>• Friend____________________________________ 1<br>• Relative___________________________________2<br>• Health worker______________________________3<br>• Social media______________________________ 4<br>• Radio_____________________________________5<br>• Television________________________________ 6<br>• News paper________________________________7<br>• Official website of Ghana Health Service_____________8<br>• Other (please specify) ________________________	☐
12	Where do you **mostly** get information on COVID-19 on daily basis?<br>**(Choose only one option)**<br>• Friend____________________________________ 1<br>• Relative___________________________________2<br>• Health worker______________________________3<br>• Social media______________________________ 4	☐

	• Radio____5<br>• Television____ 6<br>• News paper____7<br>• Official website of Ghana Health Service______________________8<br>• Other (please specify) ____	
	**SECTION C: Stigma and uptake of COVID-19 testing services**	**Response**
1	I will voluntarily test for COVID-19<br>• Very unlikely_______________1<br>• Unlikely ____________________2<br>• Undecided___________________3<br>• Likely______________________4<br>• Very likely_________________5	
2	I will advise someone to test for COVID-19<br>• Very unlikely___________________1<br>• Unlikely ______________________2<br>• Undecided___________________3<br>• Likely______________________4<br>• Very likely____________________5	
3	Do you think stigma against persons with COVID-19 is a big deal in Ghana?<br>• Yes____ 1<br>• No____2	
3	Have you ever been tested for COVID-19?<br>• Yes____ 1<br>• No____2<br>• Prefer not to disclose______________________________3<br>(If your response to this question is Yes, please proceed to answer question 4)	
4	What was the outcome of the test?<br>• Positive____1<br>• Negative____2<br>• Prefer not to disclose______________________________3<br>**(If your response to this question is Positive, please proceed to answer question 5)**	
5	Did you experience stigma?<br>• Yes____1<br>• No____2<br>**(If your response to this question is Yes, please proceed to answer questions 6-8)**	
6	Who mostly stigmatized against you?<br>• Please specify___________________________________	
7	What was your personal experience after you were diagnosed of COVID-19?<br>• Please specify______________________________________	
8	What coping strategies did you use to cope with the situation?<br>• Please specify______________________________________	
9	Do you know someone who tested positive for COVID-19?<br>• Yes<br>• No<br>**(If your response to this question is Yes, please proceed to answer questions 10-12)**	

10	Did they experience stigma?<br>• Yes<br>• No					
11	What was their experience?<br>• Please specify_______________________________________					
12	What coping strategies did they use to cope with the condition?<br>• Please specify____					
	**SECTION D: Adherence to COVID-19 prevention measures**	**Response**				
	***Instruction**: Kindly rate your level of compliance with the following* COVID-19 *preventive measures on a scale of 1 – 5 where 1 means very rarely, 2 means rarely, 3 means average, 4 means most times, 5 means all the time*					
1	Handwashing with soap under running water	1	2	3	4	5
2	Use of alcohol-based sanitizer	1	2	3	4	5
3	Wearing of face masks	1	2	3	4	5
4	Proper hand washing	1	2	3	4	5
5	Proper wearing of face mask	1	2	3	4	5
6	Stay at home	1	2	3	4	5
7	Social distancing and gathering restrictions	1	2	3	4	5
8	Eating balanced diet to boost immunity	1	2	3	4	5
9	Taking vitamin C supplements	1	2	3	4	5
10	Physical exercises	1	2	3	4	5
11	Regular intake of water	1	2	3	4	5
12	Sufficient sleep	1	2	3	4	5
	**SECTION E: Impact of the COVID-19 on livelihood**	**Response**				
	***Instruction**: Kindly rate the impact of the* COVID-19 *on your life using the scale of 1 – 5 where 1 means very high negative impact, 2 means high negative impact, 3 means minimal negative impact, 4 means no negative impact, 5 means no negative impact at all*					
1	Business or work (e.g. reduced income, laid off from work)	1	2	3	4	5
2	Education (e.g. schooling, disrupted studies, personal/career development)	1	2	3	4	5
3	Family life (e.g. family bonding, disrupted family roles, care for children and aged)	1	2	3	4	5
4	Child's education (e.g. teaching at home, babysitting)	1	2	3	4	5
5	Religious life (e.g. unable to pray effectively, cannot pray with family)	1	2	3	4	5
6	Access to healthcare (e.g. fear of going to hospital)	1	2	3	4	5
7	Food (e.g. food shortage, expensive foodstuffs)	1	2	3	4	5
8	Water (e.g. water shortage, expensive water)	1	2	3	4	5
9	Electricity (e.g. electricity cuts)	1	2	3	4	5
10	Emotional/psychological wellbeing (e.g. depression and/or anxiety)	1	2	3	4	5
	**SECTION F: Satisfaction with government response to COVID-19**	**Response**				
	***Instruction**: Kindly rate your level of satisfaction with the way government handled the COVID-19 using the scale of 1 – 5 where 1 means very dissatisfied, 2 dissatisfied, 3 means average, 4 satisfied, 5 means very satisfied*					
1	Border closure and suspension of flights	1	2	3	4	5
2	Contact tracing	1	2	3	4	5
3	Mandatory quarantine (suspected to have COVID-19)	1	2	3	4	5
4	Mandatory isolation (confirmed to have COVID-19)	1	2	3	4	5

5	Imposition of partial lockdown in Greater Accra and Greater Kumasi	1	2	3	4	5
6	Closure and reopening of tertiary and secondary schools for final year students	1	2	3	4	5
7	Closure and restrictions of churches/mosques/places of workshop	1	2	3	4	5
8	Treatment/care for Coronavirus infected persons	1	2	3	4	5
9	Provision of personal protective equipment to health workers	1	2	3	4	5
10	Provision of personal protective equipment to citizens	1	2	3	4	5
11	Public education/health communication on Coronavirus disease	1	2	3	4	5
12	Psychosocial/emotional support for Coronavirus infected persons	1	2	3	4	5
13	Managing impact of the Coronavirus disease on livelihoods	1	2	3	4	5
14	What recommendation/suggestion do you have regarding Coronavirus disease?<br>**(Please specify)**____					

	**SECTION F: Perceptions on COVID-19 Vaccine and Update**	**Response**
1	Would you participate in COVID-19 vaccine trial?<br>• Yes<br>• No	☐
2	If No to question 1 why?<br>**Please specify**_________________________________	
3	Would you advise someone (friend, relative) to participate in a COVID-19 vaccine trial?<br>• Yes<br>• No	☐
4	If No to question 3 why?<br>**Please specify**_________________________________	
5	Would you accept to be immunized with a COVID-19 vaccine?<br>• Yes<br>• No	☐
6	If No to question 5, why?<br>**Please specify**_________________________________	
7	Would you allow someone (friend, relative) to be immunized with a COVID-19 vaccine?<br>• Yes<br>• No	☐
8	If No to question 7, why?<br>**Please specify**_________________________________	
9	Would be willing to pay for COVID-19 vaccine?<br>• Yes<br>• No	☐
10	If yes to question 9, how much in GHC are you willing to pay per immunization?<br>**Please specify**_________________________________	

MANY THANKS FOR YOUR TIME
